# Bee-Inspired Healing: Apitherapy in Veterinary Medicine for Maintenance and Improvement Animal Health and Well-Being

**DOI:** 10.3390/ph17081050

**Published:** 2024-08-09

**Authors:** Jevrosima Stevanović, Uroš Glavinić, Marko Ristanić, Vladimira Erjavec, Barış Denk, Slobodan Dolašević, Zoran Stanimirović

**Affiliations:** 1 Department of Biology, Faculty of Veterinary Medicine, University of Belgrade, 11000 Belgrade, Serbia; rocky@vet.bg.ac.rs (J.S.); uglavinic@vet.bg.ac.rs (U.G.); zoran@vet.bg.ac.rs (Z.S.); 2 Small Animal Clinic, Veterinary Faculty, University of Ljubljana, 1000 Ljubljana, Slovenia; vladimira.erjavec@vf.uni-lj.si; 3 Department of Biochemistry, Faculty of Veterinary Medicine, Afyon Kocatepe University, Afyonkarahisar 03204, Turkey; bdenk@aku.edu.tr; 4 Institute for Animal Husbandry, Zemun, 11080 Belgrade, Serbia; dolasevicslobodan@gmail.com

**Keywords:** honey, propolis, bee venom, pollen, royal jelly, drone larvae, veterinary medicine

## Abstract

This review aims to present current knowledge on the effects of honey bee products on animals based on in vivo studies, focusing on their application in clinical veterinary practice. Honey’s best-proven effectiveness is in treating wounds, including those infected with antibiotic-resistant microorganisms, as evidenced in horses, cats, dogs, mice, and rats. Propolis manifested a healing effect in numerous inflammatory and painful conditions in mice, rats, dogs, and pigs and also helped in oncological cases in mice and rats. Bee venom is best known for its effectiveness in treating neuropathy and arthritis, as shown in dogs, mice, and rats. Besides, bee venom improved reproductive performance, immune response, and general health in rabbits, chickens, and pigs. Pollen was effective in stimulating growth and improving intestinal microflora in chickens. Royal jelly might be used in the management of animal reproduction due to its efficiency in improving fertility, as shown in rats, rabbits, and mice. Drone larvae are primarily valued for their androgenic effects and stimulation of reproductive function, as evidenced in sheep, chickens, pigs, and rats. Further research is warranted to determine the dose and method of application of honey bee products in animals.

## 1. Introduction

Honey bee products, used by humans for centuries, have been gathering increasing attention in the scientific and professional public due to their therapeutic potential. Their use for numerous health problems, known as apitherapy, steadily increases in humans. However, the application of bee products in veterinary medicine is much less known to the professional and general public, although evidence shows their effectiveness in treating various diseases in animals.

Apitherapy refers to the use of honey bee (*Apis mellifera*) products for the treatment or prevention of health problems in humans and animals and is considered a branch of complementary and alternative medicine. Honey bee products encompass a diverse range of substances, including those derived primarily from plants (nectar, pollen, and plant resins collected and processed by bees with their secretions into honey, bee pollen, and propolis) and those synthesized by bees from their glands (such as royal jelly and bee venom). It is less known that bee larvae, primarily drone larvae, also hold promise for health maintenance and improvement [1,2].

This work distinguishes itself through its original focus, emphasizing findings from in vivo studies, namely those conducted on animals. Furthermore, the included studies are meticulously selected, adhering to three key criteria: they must have been published within the last 15 years, reported in peer-reviewed journals, and available in full-text format.

## 2. Honey

Clinical trials have extensively investigated the effects of honey on a wide range of animals, including dogs, horses, cats, cattle, and pigs, with preclinical trials involving rats, mice, and rabbits [3]. Once overshadowed by antibiotics, medical-grade honey is regaining popularity due to its antimicrobial and pro-healing properties, making it valuable for cutaneous wound healing [4]. Honey’s best-known and proven efficacy in animals is in the treatment of burns and wounds, especially those refractory to conventional treatments [3,4,5,6,7]. Among the various types of honey, mānuka honey, derived from the *Leptospermum scoparium* shrub native to southeastern Australia and New Zealand, has proven particularly effective. The advantage of mānuka honey over other honey types is attributed to its unusually high methylglyoxal (MGO) content, considered a major antibacterial compound in mānuka honey [8]. However, there are other mechanisms of antimicrobial action characteristic of most honey types: enzymatic production of hydrogen peroxide (H_2_O_2_), high osmolarity due to high sugar concentration, low pH level, and the presence of the bee antimicrobial peptide defensin-1 [9]. In a study on horses, contaminated wounds on the hind limbs healed faster and more successfully when treated with mānuka honey gel or just mānuka honey compared to untreated control wounds [5]. However, in an experiment on mice, chestnut honey showed better efficacy than mānuka honey in terms of angiogenesis and reepithelialization on the seventh day of treatment, while after 14 days, the effect of both kinds of honey was equally effective and significantly better compared to the control [7]. Importantly, mānuka honey may be less effective in treating wounds because its high MGO content adversely affects two of the mechanisms of antimicrobial action by modifying protein components; MGO inhibits glucose oxidase (the enzyme responsible for H_2_O_2_ generation), so the accumulation of H_2_O_2_ is disabled [10], while defensin-1, after modification by MGO, loses its antibacterial activity [11].

Interestingly, MGO concentration in mānuka honey is still considered one of its main quality factors (presented as an important potency measure) incorporated in the ”Unique Mānuka Factor” (UMF™) grading system. Originally, the UMF grade reflected only the level of antibacterial activity of honey without information about the components responsible for it [9]. With the discovery of MGO and its antibacterial potency, its concentration became the key signature marker for UMF [12]; however, MGO alone cannot explain the entirety of the antimicrobial effectiveness of mānuka honey, as this honey inhibits the growth of pathogenic bacteria at concentrations well below the minimum inhibitory concentration (MIC) of MGO alone [13,14,15,16]. The relationship between the MGO content of honey and its antibacterial effect varies and depends on the bacterial species [13,14,15,16,17]. For example, the positive correlation was strong in cases of *Enterococcus faecalis* and *Escherichia coli* and moderate in the case of *Staphylococcus aureus*, but no relationship was recorded between MGO content and efficacy against *Pseudomonas aeruginosa* [17]. The latest is in accordance with the previous evidence that MGO is not solely responsible for the efficacy of mānuka honey against *P. aeruginosa* [13,14,15,16]. These findings explain the result of an earlier study in which manuka honey with a lower UMF value showed greater antimicrobial activity than honey with a higher UMF value [18]. Overall, neither the MGO level nor the UMF value represents a reliable indicator of the antibacterial effect.

**Figure 1:**
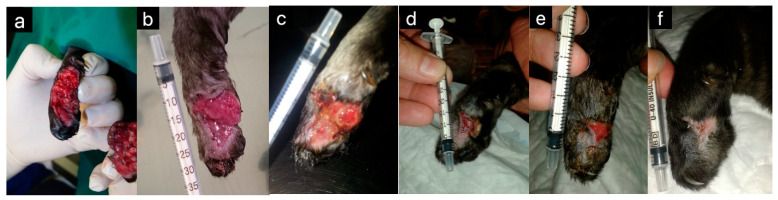
A part of the leg of a 1-year-old female was cat cut off by a lawnmower. The wound healed completely by the second intention with the use of medical honey. The sterile syringe was used as a scale for later wound size assessment. The leg on the day of presentation (**a**), 7 days (**b**), 11 days (**c**), 19 days (**d**), 24 days (**e**), and 30 days later (**f**). Medical grade honey was applied to the wound daily for the first 10 days and thereafter every 2–3 days (Original photo, V. Erjavec).

**Figure 2:**
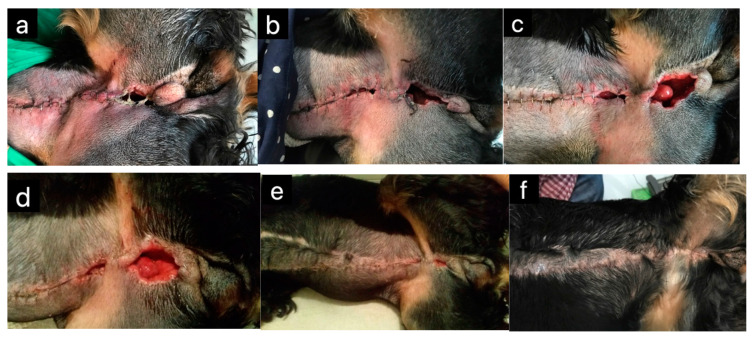
Dehiscence after unilateral mastectomy in a 12-year-old bitch: (**a**) on the day of presentation; (**b**) two days after presentation; (**c**) four days after presentation; (**d**) 14 days after presentation; (**e**) 21 days after presentation; (**f**) two months after presentation. Medical-grade honey was applied to the wound daily by the owner (Original photo, V. Erjavec).

**Figure 3:**
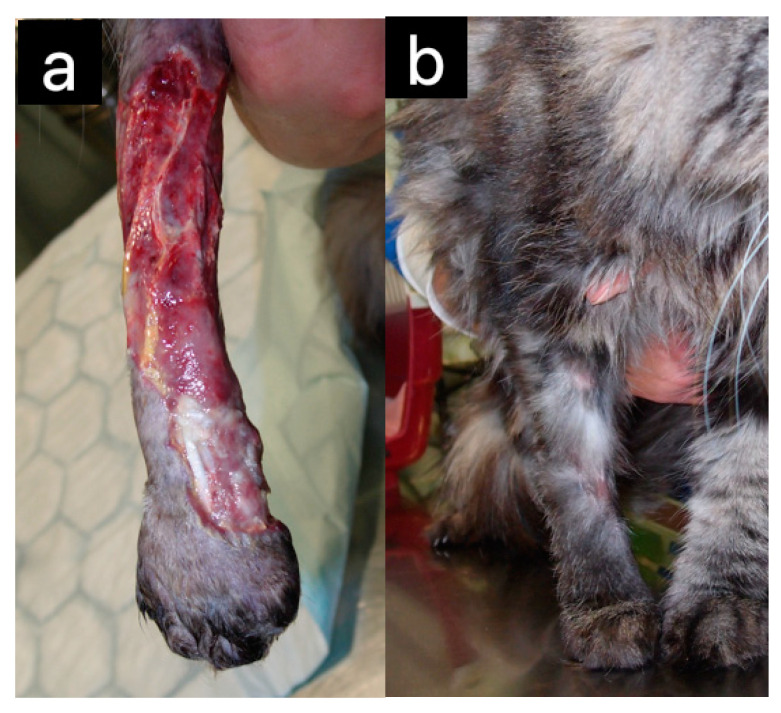
Complete skin loss across 100% of an 8-year-old female cat’s forelimb (**a**) healed completely with minimal scarring (**b**) using medical honey [6]. (Original photo, V. Erjavec).

Excellent effects in wound healing with honey were demonstrated in cats [6,19] (Figure 1—not previously published) and dogs [20,21] (Figure 2—not previously published) regardless of whether a commercial product with medical-grade honey (L-Mesitran^®^) was used [6,19,21] or raw honey was applied to the gauze and then to the wound [20]. L-Mesitran^®^ showed exceptional efficacy in treating cutaneous injuries. For instance, in a case where a cat had complete skin loss across 100% of its leg with a fractured distal ulna, the leg healed entirely, including the bone, with the regrowth of new skin and hair [6] (Figure 3).

Full-thickness skin wounds of cats treated with medical grade honey exhibited superior outcomes (reduced edema, enhanced angiogenesis, and increased fibroblast concentration) than those treated with *Hypericum perforatum*; the latter significantly improved only tissue perfusion compared to the untreated controls [4]. This is a very important result since *H. perforatum* is traditionally used for wound healing, and it achieves healing effects through the stimulation of epithelialization and granulation [22,23], collagen accumulation [22], and the expression of genes related to tissue repair and regeneration [24].

**Figure 4:**
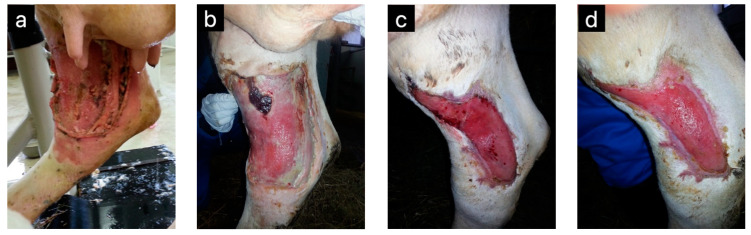
An extensive wound on a cow’s rare leg was successfully healed with raw honey, not medical-grade honey. An infected wound with necrotic material and surrounding erythema on the first day of treatment (**a**), wound covered with some necrotic tissue and pus 14 days later (**b**), the wound is almost clean, has contracted, is covered with granulation tissue (**c**) and is epithelializing from the margins (**d**) 1.5 and 2.5 months later (Original photos donated by Clinic of ruminants, Veterinary Faculty, University of Ljubljana).

An extensive leg wound in a 5-year-old female cattle was also successfully treated with raw honey, further proving honey’s remarkable healing properties, although the wound did not fully heal due to the cow being slaughtered before complete recovery (Figure 4). The wound was rinsed with tap water, covered with raw honey, and bandaged daily for the first two weeks. Thereafter, the bandage was changed, and honey was applied every second day. No antiseptics were used during the wound healing.

In addition to wound treatment, experiments on rats showed the efficacy of honey in the prevention and treatment of gastrointestinal problems, especially in the healing of gastric ulcers [25,26,27,28] and disorders related to metabolic syndrome, such as hyperglycemia, dyslipidemia, and hyperlipidemia [29,30,31,32,33].

## 3. Propolis

There is much evidence of the successful treatment of animals with propolis products. Propolis-based products have proven to be a good alternative to conventional antimicrobials, with ample evidence of successful treatment in animals. For example, propolis has shown an excellent antifungal effect in preclinical studies on laboratory mice with vulvovaginal candidiasis; mucoadhesive propolis-based gel demonstrated antifungal efficacy similar to clotrimazole cream [34], while propolis extract incorporated in mucoadhesive thermoresponsive systems showed antifungal action similar and even superior, depending on the propolis concentration, to those with nystatin [35].

The great attention of veterinarians has been attracted by the possibility of using propolis in the treatment of bovine mastitis caused by microorganisms resistant to conventional antibiotics because of the promising efficacy that propolis ethanolic extracts demonstrated against etiological agents of bovine mastitis, *Staphylococcus aureus* and *Escherichia coli*. However, that efficacy was demonstrated indirectly, e.g., in vitro, against standard strains and wild-types of *S. aureus* and *E. coli* isolated from mastitic milk [36], against *S. aureus* cultivated in complex media or milk [37], and against damages induced by *S. aureus* and *E-coli* in bovine mammary epithelial cells [38]. However, negative effect of propolis on mammary tissue was also reported, as it reduced the viability of bovine mammary gland explants [36]. Thus, before the introduction of propolis into the veterinary practice of mastitis treatment in cattle, clinical studies should confirm the effectiveness, dosage, and safety of propolis use in mastitis control.

The antiviral effects of propolis have been evidenced in some in vivo studies, mostly against herpes simplex viruses HSV-1 and HSV-2 [39,40]. The antiparasitic effect has been evidenced in animal models for some types of propolis. After oral administration, propolis extracts effectively decreased the infection level of the microsporidian parasite *Nosema ceranae* in honey bee intestine [41,42,43]. Propolis extracts were also highly effective against other protistan parasites, such as *Trypanosoma brucei* [44], *Plasmodium falciparum*, *P. berghei* [45], and *P. chabaudi* [46], as evidenced in experiments on rats applying doses 400 and 600 mg/kg against *T. brucei* [44] and much lower doses against *Plasmodium* sp. in mice [45,46]. The antiparasitic properties of propolis have also been recorded against helminths, such as against *Schistosoma mansoni*. A single dose of propolis (400 mg/kg) administered to mice significantly reduced the burden of worms and the number of eggs, both immature and those in feces [47].

Exceptional success in wound healing with propolis products has been evidenced in several animal species [48]. In rats, all tested formulations of propolis gel, containing 1.2%, 2.4%, and 3.6% *w*/*v* (dry matter) of propolis, showed good activity in healing skin lesions, and the best results were achieved in the group treated with a gel containing 3.6% propolis [49]. In dogs, propolis paste 30% significantly reduced the healing time of wounds due to enhanced reepithelization and contraction of wounds [50]. Healing burn wounds with ointments containing propolis was very successful in pigs [51,52]. The healing effect is explained by the prevention of fibronectin biosynthesis and its degradation in the wound area [51], as well as by the broad-spectrum antibacterial activity, acceleration of neoangiogenesis and epithelialization [52].

Interestingly, propolis, as an aqueous or ethanolic extract, was shown to be effective as an adjuvant in veterinary vaccines and even superior to traditional vaccine adjuvants [53]. For example, propolis significantly increased antibody production when added to canine parvovirus and canine coronavirus vaccines [54], a vaccine against porcine parvovirus [53], and a vaccine against the bacterium *Aeromonas salmonicida*, the cause of furunculosis in fish [55].

Hydroalcoholic extracts of red propolis have shown gastroprotective properties in rats. The basis of this effect was a significant inhibition of the development of acute ulcers, a significant decrease in the volume of gastric secretion, and a significant increase in the production of gastric mucus, as well as antioxidant, and anti-*Helicobacter pylori* activities [56].

As added to broiler feed, even at low doses (10 mg/kg), propolis effectively protected the liver and blood vessels by inhibiting the formation of pathological lesions; with an increase in the dose to 50 mg/kg, the positive effects were even better [57].

Propolis has also been found to have anticancer potential [58] and alleviate the toxic effects of chemotherapy agents [59]. The antitumor activity of propolis observed in in vivo experiments (on rats and mice) indicates its potential application in the treatment of oncological cases in veterinary medicine [58,59,60].

Propolis is effective in healing numerous inflammatory and painful conditions in the oral cavity, such as candidiasis, gingivitis, periodontitis, ulcers, pulp mummification, supragingival plaque, caries, herpes, wounds after surgical interventions, and cancers, as evidenced in experiments on laboratory animals [61,62,63,64]. Besides, employing propolis for tooth storage before reimplantation in healthy mixed-breed dogs proved to be effective in enhancing the success rate of the procedure [65]. It is also worth noting the excellent effectiveness of topically applied propolis ear drops in otitis externa in dogs [66] and propolis eye drops in corneal wound healing in rats [67].

When administered as a dietary supplement to alloxan-induced diabetic mice, propolis preparations mitigated hepatotoxicity and nephrotoxicity by reducing oxidative stress and minimizing the deleterious effects of free radicals on tissue [68].

Propolis achieves therapeutic properties thanks to its complex composition, but primarily to numerous phenolic compounds, of which flavonoids, phenolic acids, and esters are the most biologically active [69,70]. For propolis activities, many underlying mechanisms have been discovered and reviewed [69,70,71]. For example, the antimicrobial effect of propolis is based on stimulating macrophages to activate Th1 cells, which enhances the cellular immune response (release of cytokines that are essential coordinators of the immune response to intracellular pathogens); in some microorganisms, propolis disrupts the permeability of the cell membrane leading to leakage of cellular components [71]. The anti-inflammatory activity of propolis is accomplished by inhibiting cyclooxygenase and prostaglandin biosynthesis, neutralizing free radicals, reducing cytokines, nitric oxide synthesis, and immunosuppression [70]. Various mechanisms are involved in the anticancer effect of propolis, among which are those already mentioned, including its powerful antioxidant and immunomodulatory potential, as well as its ability to stimulate cell cycle arrest, induce apoptosis, inhibit angiogenesis and metastasis of tumors, inhibit specific oncogene signaling pathways, inhibit glucose uptake and metabolism in the cancer cell, and possibly participate in epigenetic regulation [71].

## 4. Bee Venom (Apitoxin)

Bee venom has been used since ancient times in the treatment of rheumatoid arthritis, and its success in therapy lies in its anti-inflammatory and antinociceptive effects, reducing swelling and pain, as evidenced by numerous preclinical and clinical studies [72,73,74]. Melittin, the dominant component of bee venom (≥50% *w*/*w* of bee venom), is responsible for its anti-inflammatory effect and also exhibits anticancer potential [75,76]. For melittin, as well as other components of bee venom (phospholipase A2 and apamin), a neuroprotective effect has been demonstrated, explaining the positive effects of bee stings on neurodegenerative diseases such as Parkinson’s disease, multiple sclerosis, and intervertebral disc degeneration recorded in experiments on dogs [77] and mice [78,79,80]. In experimental animals, bee venom was effective in the therapy of neuropathies caused by nerve injuries or chemotherapeutics [81,82,83].

Bee venom acupuncture has proven useful in the case of a dog diagnosed with idiopathic facial paralysis; such a therapy led to a gradual improvement of clinical signs, and complete recovery of sensory and neurological facial signs in a dog was noted after eight weeks [84].

Thanks to its antioxidant potential, bee venom improves reproductive performance, immune response, and general health status, as demonstrated in rabbits [85,86]. Antioxidant, anti-inflammatory, and anti-apoptotic mechanisms were underlying the gastroprotective effect of bee venom in an experiment on mice [87]. When added as a supplement to food or water, bee venom has improved feed conversion and increased body weight in broiler chickens without adverse side effects [88,89], while administering bee venom through a live bee sting or by injection positively affected the growth, survival, and immunity of young pigs [90]. In vivo studies on dogs [91], broiler chickens [92], mice [93], and pigs [94] have also shown that bee venom acts as an immunoprophylactic agent, as well as having antibacterial, antifungal, and antiviral effects. The positive therapeutic effect of melittin in animals infected with methicillin-resistant *Staphylococcus aureus* (MRSA) opens up the possibility of its use in treating MRSA infections [95]. Finally, the antidiabetic potential of bee venom was studied on laboratory rats [96,97]. A single-dose injection (0.5 mg/kg) can be considered an initial step in demonstrating its antidiabetic effects [97]. Additionally, the selection of solvent for the use of dry bee venom in injectable and/or topical forms is crucial. This is because the degradation/dysfunction of a specific peptide and the more complex enzymatic bioactivity of the main components in the venom should be prevented, thereby preserving the desired effectiveness of the treatment. To ensure optimal antioxidant activity, we recommend to dissolve the venom in physiological saline [98].

## 5. Pollen

Due to its exceptional nutritional composition, bee-collected pollen (known as “bee pollen”) is considered a “functional food” and is most commonly recommended as a dietary supplement [99]. The potential of bee pollen in animal husbandry was mainly investigated in terms of its effect on growth performance, meat performance and slaughter yield. Adding bee pollen or its ethanolic extract to poultry feed in a range of 400–800 mg/kg feed effectively stimulated animal growth and improved broiler gut microflora [100]. However, this supplementation did not significantly improve meat performance and slaughter yield [101]. Even a ten-times higher concentration of bee pollen (7.5–20 g/kg feed) given to broilers did not affect slaughter yield despite significant improvement in the growth performance, the immune response [102,103,104], and the microbiological composition of the intestine [104].

The medicinal effects of bee pollen have been demonstrated only in treating benign prostatic hyperplasia and inflammation [105,106,107] and diabetes-induced testicular dysfunction [108] in experiments on rats.

From the above, one gets the impression that the evidence of the apitherapeutic effects of pollen on animals is scarce. This may be due to the poor bioavailability of pollen nutrients (due to the strong protective layers of pollen grains) and the extreme susceptibility of pollen to microbial spoilage. That is why it is necessary to work on the development of new technologies that will prevent pollen spoilage, protect sensitive pollen compounds, and ensure a higher degree of accessibility [99].

## 6. Royal Jelly

Very little data exists on the clinical effects of royal jelly. Here, we show only those that meet the criteria set for this review paper. In in vivo experiments (on laboratory rats), royal jelly was shown to protect the body from the toxic effects of chemotherapy, including nephrotoxicity, hepatotoxicity, and pulmonary fibrosis [109,110,111]. The anticancer potential of royal jelly has been demonstrated in experiments on mice [112,113]. Royal jelly is also attributed to anti-aging effects, supported by scientific evidence obtained in experiments on mice [114]. In immature rats, royal jelly has been found to promote folliculogenesis and increase ovarian hormones [115], and in ovariectomized rats, it improved bone strength [116] and showed potential in the prevention of osteoporosis [117]. It has been shown in rabbits to alleviate neurological disorders by increasing estrogen levels and the activity of the cholinergic and antioxidative systems while reducing cholesterol and restoring the autonomic nervous system [118]. Interestingly, royal jelly showed beneficial effects related to male fertility and reproductive success in rats [119,120,121], mice [122,123], and rabbits [124,125]. Bioactive components responsible for royal jelly biological activities are fatty acids (e.g., 10-hydroxy-2-decenoic acid), free amino acids, peptides, proteins, and phenolic compounds, among which the most numerous are flavonoids [126].

## 7. Drone Larvae

It is well known that animals have been eating honey bee larvae to provide significant amounts of energy and supplement their protein needs [2,127]. However, due to its importance for the bee colony, worker brood should not be taken, while drone brood can be used in cases where it is obtained as a by-product of the usual beekeeping procedure known as ’drone brood removal’ aimed at controlling the *Varroa destructor* mite. This is a biotechnical procedure in which beekeepers insert a special ’trap frame’ (frame with comb cells of a size corresponding to drone brood) into the hive to attract *V. destructor* mites (because drone brood is eight times more attractive to them than worker brood) [128]. When removing the ’trap frames’ from the hive, the entire drone brood is discarded. This product is generally considered waste and remains unused despite its exceptional chemical composition and high nutritional value (due to the presence of proteins, fatty acids, vitamins, hormones, and antioxidants) [127], as well as pharmacological properties [2] and potential that justify its use in maintaining health. In veterinary medicine, drone larvae are primarily valued for their androgenic effects and positive impact on reproductive performance, fertility, and/or productivity. Androgenic effects have been demonstrated in various animals (laboratory and farm animals), and the studies almost always used drone larvae homogenate (DLH), either freshly made or in the form of the commercial preparation ‘Apilarnil’ invented by Nicolae Iliesiu in 1981 in Romania. ’Apilarnil’ is made from drone larvae through a process that includes homogenization, filtration, and lyophilization [2]. For example, the androgenic effects of DLH have been demonstrated in rams when given as a dietary supplement (at doses of 10, 15, and 20 mg/kg body weight); all doses improved ram reproductive functions, reflected in increased offspring numbers. The optimal effect on sperm quality was achieved with a dose of 15 mg/kg of body weight administered once daily, which resulted in a 30.4% increase in ejaculate volume, a 14.3% increase in sperm concentration in ejaculate, and a sperm motility score of 9.2/10 points [129]. The androgenic effect of DLH in castrated laboratory rats was evidenced through a significant increase in the expression of Spot14-like androgen-inducible protein (SLAP) in rat prostate [130]. In male broilers, food supplementation with DLH during the growth period also led to androgenic effects; after 20 days of administration of DLH (4 g per broiler per day), secondary sexual characteristics (comb size, wattle size, and aggression) were more pronounced than in the control group [131]. When DLH was added to the feed of broilers aged 28 to 55 days, at doses of 2.5 and 7.5 g per individual, it also stimulated the development of secondary sexual characteristics and sexual maturation in males (increased testis weight, increased testosterone concentration, and comb size), but also led to a decrease in blood glucose and cholesterol levels and a reduction in fear [132]. Feed supplemented with DLH in doses of 2.5 and 7.5 g per individual did not stimulate the development of secondary sexual characteristics in female broilers, and anabolic effects were absent regardless of the sex of the broilers [132].

In females, the effect of DLH is different; it exhibited anabolic and actoprotective effects in young pigs when administered as a dietary supplement. Namely, given to gilts at a concentration of 25 mg of dry matter per 1 kg of food, DLH significantly increased in production parameters (live weight, average daily gain, and slaughter yield) [133]. Besides, DLH significantly affected hormone levels (increased cortisol and decreased testosterone), whereby a significantly better effect was achieved when selenopyran was introduced into the food along with DLH at a concentration of 1.2 mg per 1 kg of food [133]. An anabolic effect was also recorded in another experiment on gilts, where DLH was added to the feed (25 mg/kg of forage) for 180 days [134]; the results motivated the authors to suggest DLH as a valuable dietary additive in livestock farming for increasing meat production. However, in the same experiment, DLH significantly affected folliculogenesis in the ovaries; it stimulated the early stages of folliculogenesis but caused a decrease in the size of Graafian follicles with signs of atresia in the final stage of follicular development, along with changes in the expression of growth factors GDF9 and BMP15 responsible for proper follicle development and ovulation. In another study, supplementary feeding with 0.5 g of DLH daily enhanced the reproductive function of gilts during puberty by reducing the time to their first estrous cycle for artificial insemination [135]. Therefore, care should be taken when treating gilts with DHL (can be given to those intended for slaughter but not to those planned for reproduction).

Finally, in experimental rats, DLH showed neuroprotective potential and therapeutic potential in sepsis. Namely, it prevents the apoptosis of Purkinje cells by lowering the levels of pro-inflammatory cytokines (IL-6, TNF-α, IL-1β) that are elevated during sepsis and thus prevents sepsis-induced apoptosis in the brain [136]. It also protects the liver from lipopolysaccharide (LSP)-induced damage by reducing tissue damage, inhibiting the TLR4/HMGB-1/NF-κB signaling pathway, and protecting liver cells from DNA damage and oxidative stress [137].

## 8. Precautions and Final Remarks

It is important to state that products from the honey bee hive can be contaminated, both with chemicals (primarily acaricides used by beekeepers to control the honey bee mite *Varroa destructor*, but also with other pesticides commonly used in agriculture) and other environmental pollutants [138,139]. Fat-rich products such as wax and propolis, as well as hive air, are particularly prone to contamination [140,141,142]. Therefore, for the protection of health, strength, and immunity of honeybee colonies, it is advisable to either utilize products derived from natural sources, such as plants, algae, and fungi [143,144,145,146,147,148,149,150,151,152,153], or employ traditional beekeeping methods that minimize stress, reduce pathogens exposure, and negate the need for chemical treatments [154,155].

Finally, it should be emphasized that apitherapy in veterinary practice can only be applied after examination by a licensed veterinarian. Additionally, when using any bee product, care should be taken regarding the possibility of allergic reactions, though they are less commonly reported compared to humans. If an allergic reaction such as envenomation with honey bee venom is suspected, prompt veterinary attention should be sought to manage symptoms and prevent further complications [156,157].

Apitherapy has great potential for application in veterinary medicine, but officially, it can only be used as a complementary method of treatment. Growing evidence underscores the value of apitherapy in promoting animal health and vitality (Table 1). This leads to an increasing interest of animal owners and veterinarians in using bee products. However, they must recognize the potential risks associated with apitherapy, including potentially fatal anaphylactic reactions in certain cases [158,159]. However, further preclinical and clinical trials are necessary to comprehensively understand the basic mechanisms of action of bee products and determine the optimal doses and methods of their application in animals.

## Figures and Tables

**Table 1:** The effects of honey bee products are demonstrated in vivo.

Honey Bee Product	Animal	Effect	Reference(s)
Honey	Horses	Wound healing	[5]
	Cats	Wound healing	[4,6,19]
	Dogs	Wound healing	[20,21]
	Mice	Wound healing	[7]
	Rats	Gastroprotective effect	[25]
		Gastric ulcer healing	[26,27,28]
		Hypoglycemic and antioxidant effects	[29]
		Protection of pancreas	[30]
		Cardioprotective effects	[31]
		Antidiabetic effect	[32]
		Anti-atherogenic effect	[33]
Propolis	Mice	Antifungal efficacy on vulvovaginal candidiasis	[34,35]
		Antiparasitic (antimalarial activity) against *Plasmodium chabaudi*	[46]
		Antiparasitic (antimalarial) effect against *Plasmodium falciparum* and *P. berghei*	[45]
		Antiparasitic properties against *Schistosoma mansoni*	[47]
		Anticarcinogenic potentials	[60]
		Mitigating hepatotoxicity and nephrotoxicity by reducing oxidative stress	[68]
	Honey bees	Antiparasitic effect against *Nosema ceranae*	[41,42,43]
	Rats	Antiparasitic effect against *Trypanosoma brucei* and *T. congolense*	[44]
		Wound healing	[49]
		Anticarcinogenic potential	[58]
		Gastroprotective properties due to anti-oxidant and anti-*Helicobacter pylori* activities	[56]
		Protective role during the initial phases of lingual carcinogenesis	[61]
		Antimicrobial activity against mutans streptococci, Caries prevention	[62]
		Anticaries effects	[63]
		Chemopreventive and gastroprotective effects	[64]
		Corneal wound healing	[67]
	Dogs	Wound healing	[50]
		Storage medium (on teeth replantation)	[65]
		Antimicrobial effects in otitis externa	[66]
	Pigs	Burn wounds healing	[51,52]
	Chickens	Protective effect on liver and kidney	[57]
Bee venom	Dogs	Anti-inflammatory and analgesic effects	[77]
		Healing effect on *Malassezia*-related otitis externa	[91]
		Healing effect on facial paralysis	[84]
	Mice	Neuroprotective effect in Parkinson’s disease	[78]
		Neuroprotective effect in multiple sclerosis	[79]
		Antiviral efficacy against a broad panel of viruses	[93]
		Neuroprotective effects	[80,81]
	Rabbits	Positive impact on reproductive performance and immune response of male individuals	[85]
		Improvement of reproductive traits (sexual-stimulant), immune response, and health	[86]
	Chickens	Growth promoter	[88,89]
		Immunoprophylactic effects	[92]
	Pigs	Promotion of antibody production and viral clearance in PRRS virus infection	[94]
		Positive impact on growth, survival, and immunity	[90]
	Rats	Gastroprotective effect	[87]
		Antidiabetic effect	[96,97]
		Analgesic effect	[83]
		Antibacterial activity against *Staphylococcus aureus*	[82]
Pollen	Rats	Anti-inflammatory and protective effects in prostatitis treatment	[105]
		Healing effects in prostate hyperplasia and inflammation	[106,107]
		Protective role in diabetes-related glycemic control problems and sexual dysfunctions in male individuals	[108]
	Chickens	Positive effect on gut microflora colonization	[100]
		Growth promoter	[101]
		Improvement of growth performance and immune status	[102]
		Improvement in weight gain and food conversion rate	[103]
		Positive effect on daily gain, feed conversion, and microbiological composition of intestine	[104]
Royal jelly	Rats	Protection of liver and kidneys during chemotherapy	[109]
		Nephroprotective effect	[110]
		Anti-fibrotic effect against pulmonary fibrosis	[111]
		Stimulation of folliculogenesis and secretion of steroid hormones	[115]
		Improvement of bone strength after ovariectomy	[116]
		Prevention of osteoporosis after ovariectomy	[117]
		Improvement of fertility and reproductive success in males	[119,120,121]
	Mice	Antioxidant, immunomodulatory and anticancer effects	[112]
		Apoptotic, antioxidant, anti-inflammatory and anticancer effects	[113]
		Anti-aging effect	[114]
		Improvement of fertility and reproductive success in males	[122,123]
	Rabbits	Alleviation of neurological disorders after ovariectomy	[118]
		Improvement of fertility and reproductive success in males	[124,125]
Drone larvae	Sheep	Stimulation of reproductive function in rams	[129]
	Rats	Androgenic effect in castrated males	[130]
		Liver-protective effects	[137]
		Neuroprotective effect	[136]
	Broilers	Androgenic effects	[131]
		Androgenic effects, Decrease in blood glucose and cholesterol levels	[132]
	Pigs	Anabolic effects (increase in production parameters) in females	[133]
		Anabolic effect in females	[134]
		Improvement of fertility; stimulation of reproductive function (by reducing the time to the first estrous cycle for artificial insemination)	[135]
